# Complete Genome Sequence of Klebsiella pneumoniae Jumbo Phage Miami

**DOI:** 10.1128/MRA.01404-20

**Published:** 2021-01-28

**Authors:** Daniel Mora, Lauren Lessor, Tram Le, James Clark, Jason J. Gill, Mei Liu

**Affiliations:** a Center for Phage Technology, Texas A&M University, College Station, Texas, USA; Queens College

## Abstract

Bacteriophage Miami infects Klebsiella pneumoniae, a Gram-negative pathogen that is becoming an increasing threat to public health due to its multidrug resistance. Here, we describe the annotation of the 253,383-bp jumbo phage Miami genome sequence and its similarity to other myophages.

## ANNOUNCEMENT

Klebsiella pneumoniae is a Gram-negative, encapsulated, nonmotile bacterium that can be found in soil, in water, and on human mucosal surfaces (1). Over the last few decades, K. pneumoniae has gained a reputation for being an opportunistic nosocomial multidrug-resistant (MDR) pathogen (1). The reputation of K. pneumoniae has guided scientific efforts to develop alternative methods to treat this bacterium, largely due to the lack of available antibiotics that effectively treat MDR K. pneumoniae infections (2). The reimplementation of bacteriophages as an alternative treatment method in the clinical setting has led to a revival of bacteriophage therapeutics (3). The genome annotation of K. pneumoniae myophage Miami is detailed in this report.

Bacteriophage Miami was isolated from a wastewater treatment plant in Houston, TX, against clinical K. pneumoniae strain 44809 (GenBank accession no. NDDK01000000), which has sequence type 1502, bioinformatically predicted using the Kaptive database (4, 5). Host bacteria were cultured on tryptic soy broth at 37°C with aeration, and phage isolation and propagation were performed using the soft-agar overlay method (6). DNA was purified following a previously described protocol using the Promega Wizard DNA cleanup system (7). DNA fragmentation and library preparation were done following the manufacturer’s protocol to generate inserts with an average size of 300 bp using a TruSeq Nano kit (Illumina) and sequenced on an Illumina iSeq 100 instrument with paired-end 150-bp reads using iSeq 300-bp chemistry. The resulting 802,622 reads were quality controlled with FastQC (www.bioinformatics.babraham.ac.uk/projects/fastqc) and trimmed with FASTX-Toolkit v0.0.14 (http://hannonlab.cshl.edu/fastx_toolkit/). SPAdes v3.5.0 was then used for *de novo* assembly with default settings to generate a raw contig at 37.6-fold coverage (8). Because the sequences of the raw contig ends are often not accurate (either with redundant sequences or missing sequences), the end sequences were corrected by PCR to amplify the end region (with the primers TTTATGCCGTCTCCCCCTCT and GAGTAGCCAGCGACACAGAT facing off the ends of the contig) and Sanger sequencing of the product. Identification of genes was conducted using MetaGeneAnnotator v1.0, GLIMMER v3.0, and ARAGORN v2.36 (9–11). Following the gene identification, gene function was predicted using InterProScan v5.33, a BLAST v2.9.0 search against the NCBI nonredundant (nr) and Swiss-Prot databases (12–15), and TMHMM v2.0 with default settings. The genome-wide DNA sequence similarity of Miami was calculated using progressiveMauve v2.4 (16). The entirety of the genome analysis of phage Miami was performed via the CPT Galaxy and Web Apollo interfaces (17–19).

Miami possesses a 253,383-bp genome with a coding density of 94% and 300 predicted protein-coding genes, only 47 of which have a predicted function. The genome G+C content is 43.9%, which is lower than the average G+C content of K. pneumoniae (52.7%). As is common with “jumbo” phages, Miami is phylogenetically distant from other known phages, sharing only 124 similar proteins with *Erwinia* phages Rising Sun and Joad (GenBank accession no. MF459646 and MF459647, respectively), determined by BLASTp (E, <0.001). Miami also shares 105 proteins similar to those in *Vibrio* phages 2 TSL-2019, USC-1, Aphrodite1, and 5 TSL-2019, which are classified in the genus *Aphroditevirus* (MK368614, MK905543, MG720308, and MK358448, respectively). progressiveMauve analyses determined that Miami shares the highest nucleotide identity with *Erwinia* phage Rising Sun at 11.4%.

### Data availability.

The genome sequence of phage Miami was deposited under GenBank accession no. MT701590 and BioSample accession no. SAMN14609641. The BioProject accession no. is PRJNA222858, and the SRA accession no. is SRR11558348.
